# Validation of a computational phenotype for finding patients eligible for genetic testing for pathogenic *PTEN* variants across three centers

**DOI:** 10.1186/s11689-022-09434-0

**Published:** 2022-03-23

**Authors:** Cartik Kothari, Siddharth Srivastava, Youssef Kousa, Rima Izem, Marcin Gierdalski, Dongkyu Kim, Amy Good, Kira A. Dies, Gregory Geisel, Hiroki Morizono, Vittorio Gallo, Scott L. Pomeroy, Gwenn A. Garden, Lisa Guay-Woodford, Mustafa Sahin, Paul Avillach

**Affiliations:** 1 Department of Biomedical Informatics, Harvard Medical School, Boston, MA 02115 USA; 2 Department of Neurology, Rosamund Stone Zander Translational Neuroscience Center, Boston Children’s Hospital, Harvard Medical School, Boston, MA 02115 USA; 3 Division of Neurology, Children’s National Hospital, Washington, DC 20010 USA; 4 Department of Genomics and Precision Medicine, The George Washington University School of Medicine and Health Sciences, Washington, DC 20052 USA; 5 Division of Biostatistics and Study Methodology, Children’s National Research Institute, Silver Spring, MD 20910 USA; 6 Division of Biostatistics and Study Methodology, Children’s National Hospital, Washington, DC 20010 USA; 7 Institute for Translational Health Sciences, University of Washington, Seattle, WA 98195 USA; 8 Center for Genetic Medicine Research, Children’s National Hospital, Washington, DC 20010 USA; 9 Department of Genomics and Precision Medicine, The George Washington University School of Medicine and Health Sciences, Washington, DC 20052 USA; 10 Center for Neuroscience Research, Children’s National Research Institute, Children’s National Hospital, Washington, DC 20010 USA; 11 Department of Neurology, Boston Children’s Hospital, Harvard Medical School, Boston, MA 02115 USA; 12 Department of Neurology and Center on Human Development and Disability, University of Washington, Seattle, WA 98195 USA; 13 Department of Neurology, University of North Carolina, Chapel Hill, NC 27599 USA; 14 Center for Translational Research, Children’s National Hospital, Washington, DC 20010 USA

**Keywords:** Rare disease, Genetic disease, Computational phenotype, Electronic health records, Autism

## Abstract

**Background:**

Computational phenotypes are most often combinations of patient billing codes that are highly predictive of disease using electronic health records (EHR). In the case of rare diseases that can only be diagnosed by genetic testing, computational phenotypes identify patient cohorts for genetic testing and possible diagnosis. This article details the validation of a computational phenotype for *PTEN* hamartoma tumor syndrome (PHTS) against the EHR of patients at three collaborating clinical research centers: Boston Children's Hospital, Children's National Hospital, and the University of Washington.

**Methods:**

A combination of billing codes from the International Classification of Diseases versions 9 and 10 (ICD-9 and ICD-10) for diagnostic criteria postulated by a research team at Cleveland Clinic was used to identify patient cohorts for genetic testing from the clinical data warehouses at the three research centers. Subsequently, the EHR—including billing codes, clinical notes, and genetic reports—of these patients were reviewed by clinical experts to identify patients with PHTS.

**Results:**

The *PTEN* genetic testing yield of the computational phenotype, the number of patients who needed to be genetically tested for incidence of pathogenic *PTEN* gene variants, ranged from 82 to 94% at the three centers.

**Conclusions:**

Computational phenotypes have the potential to enable the timely and accurate diagnosis of rare genetic diseases such as PHTS by identifying patient cohorts for genetic sequencing and testing.

**Supplementary Information:**

The online version contains supplementary material available at 10.1186/s11689-022-09434-0.

## Background

### Computational phenotypes for rare diseases

Computational phenotypes [1, 2] are combinations of clinical billing and diagnostic codes that are highly indicative of disease and are identified either manually [3–6] or by machine learning algorithms from patients’ electronic health records (EHR) [7–13]. The Phenotype Knowledge Base (PheKB, http://phekb.org) lists computational phenotypes for over fifty diseases with more submissions under review. The identification of computational phenotypes or “phenotyping” is reliant on the availability of large patient cohorts, common knowledge about disease symptoms, and standardized codes for clinical diagnoses, procedures, and lab tests.

In the case of rare diseases [14] where patient populations are small, knowledge of the breadth of patient symptoms can be limited [15–17], and diagnosis may depend on genetic testing, computational phenotypes serve two different purposes. They (1) enable the identification of patients who may have a suspected genetic disorder and who could be referred for appropriate confirmatory genetic testing and (2) reveal previously undiscovered patterns of clinical comorbidities that can enhance the clinical characterization of the disease [9].

### *PTEN* hamartoma tumor syndrome

*PTEN* hamartoma tumor syndrome (PHTS) [18] is a rare genetic disorder, which encompasses four major clinically distinct syndromes: (a) Cowden syndrome (CS; OMIM: 615107, 615108, 615109) [19, 20], (b) Bannayan-Riley-Ruvalcaba syndrome (OMIM: 158350) [21], (c) Proteus syndrome (OMIM: 176920) [22], and (d) Proteus-like syndrome. All the disorders are associated with germline pathogenic variants of the phosphatase and tensin homolog (*PTEN*) tumor suppressor gene (NCBI Gene ID: 5728; HGNC ID: 9588), located on the long arm of chromosome 10. The clinical manifestations of PHTS are diverse and constitute a wide spectrum of neurological and developmental characteristics (e.g., macrocephaly, intellectual disability, autism spectrum disorder, attention deficit hyperactivity disorder, and anxiety), gastrointestinal manifestations (e.g., gastrointestinal polyps), vascular and nonvascular skin findings (e.g., arteriovenous malformations, hemangiomas, trichilemmomas, acral keratoses, lipomas, fibromas), and oncological concerns (e.g., increased risk for various cancers including thyroid cancer, breast cancer, colorectal cancer, renal cancer, endometrial cancer, and melanoma) [18]. Some of these symptoms in isolation (such as thyroid cancer) may be found in the general population. Because of this, patients may be undiagnosed and thus do not benefit from available cancer surveillance strategies. The timely recognition of the phenotypic patterns typical to PHTS is therefore critical to patient outcomes [18].

The definitive diagnosis of PHTS is based on detection of a pathogenic germline variant in the *PTEN* gene. The presence of specific clinical features may pinpoint a need for molecular testing. In 2011, Cleveland Clinic established criteria for pathogenic *PTEN* variant screening in children (https://www.lerner.ccf.org/gmi/ccscore/documents/pediatric_criteria.html; hereon referred to as Cleveland Clinic criteria for PTEN testing or Cleveland Clinic criteria) [23]. The objective of this study is to determine the effectiveness of a computational phenotype of the Cleveland Clinic criteria in finding patients who need to be genetically tested for pathogenic *PTEN* variants, using data from EHR.

### Outline

In this paper, we describe a cross-institutional initiative among three participating clinical research centers: (a) Boston Children’s Hospital (BCH), (b) Children’s National Hospital (CNH), and (c) the University of Washington (UW), to evaluate the predictive power of a computational phenotype for PHTS in identifying patients requiring genetic testing for diagnosis of *PTEN* syndrome. This initiative was coordinated by the Intellectual and Developmental Disabilities Research Centers at each of the institutions (IDDRC, https://www.iddrc.org/).

## Methods

We used a workflow adopted for the evaluation of the predictive power of a computational phenotype for PHTS (Fig. S1).

### Data

The data used in this study are complete patient electronic health records (EHR)—comprising clinical notes, genetic reports, and billing codes—sourced from the clinical data warehouses at the three centers: BCH, CNH, and UW. The patient cohorts were identified by querying the clinical data warehouses for patients with the criteria in Table 1.

**Table 1:** Cleveland Clinic criteria for identifying pediatric patients who would benefit from *PTEN* sequencing

1. **Macrocephaly (≥ 2 Standard Deviations from Mean)** **AND** **2. At least one of the following four additional criteria** **A. Autism or Developmental Delay** **B. Dermatologic features, including lipomas, trichilemmomas, oral papillomas, or penile freckling** **C. Vascular features, such as arteriovenous malformations or hemangiomas** **D. Gastrointestinal Polyps**	

The criteria above were proposed by a team of researchers at Cleveland Clinic after evaluation of a cohort of pediatric individuals with *PTEN* mutations [23] and will be referred to henceforth in this paper as the Cleveland Clinic pediatric clinical criteria or simply as the Cleveland Clinic criteria. A clinical expert identified the billing codes from the International Classification of Diseases versions 9 and 10 (ICD-9 and ICD-10) [24, 25] that correspond to the conditions in the Cleveland Clinic criteria. The list of the identified billing codes can be found in Table S1. The sizes of the patient cohorts identified by the Cleveland Clinic criteria at the three centers are shown in Table 2.

**Table 2:** Number of patients identified as having met Cleveland Clinic criteria using informatics approach across the three sites

	Boston Children’s Hospital	University of Washington	Children’s National Hospital
	From January 2001 to October 2019	January 2001 to August 2019	From August 2012 to October 2019
Total patients with at least one ICD-9 or ICD-10 code available for review	1.78 M	2.7 M	2.11 M
Number of patients identified by informatics approach as having met Cleveland Clinic criteria	1,215	104	481
Number of patients identified by informatics approach as having met Cleveland Clinic criteria whose charts were manually reviewed	396	94	481
Number of patients who had any genetic testing (from among those whose charts were manually reviewed)	204	29	227
Number of patients who had any genetic testing which included *PTEN* sequencing and/or deletion duplication analysis (from among those whose charts were manually reviewed)	90	17	43
Number of patients who satisfied Cleveland Clinic criteria after human review (from among those whose charts were manually reviewed)	371	77	438
Number of patients with pathogenic or likely pathogenic variant in *PTEN* (from among those whose charts were manually reviewed)	14	0	13
Yield of the informatics approach in identifying patients who meet Cleveland Clinic criteria (from among those whose charts were manually reviewed)	371/396 (94%)	77/94 (82%)	438/481 (91%)
Number of patients with PHTS divided by number of those identified as having met Cleveland Clinic criteria using informatics approach whose charts were manually reviewed	3.54% (14/396)	0 (0/94)	2.70% (13/481)
Number of patients with PHTS divided by number of those identified as having met Cleveland Clinic criteria using informatics approach and who also had genetic testing which included detection of *PTEN* variants (from among those whose charts were manually reviewed)	15.6% (14/90)	0% (0/17)	30.2% (13/43)

The Institutional Review Board (IRB) at Boston Children’s Hospital served as the single IRB with reliance agreements and approved this study (P00029725). The clinical data warehouses at the three participating centers were queried for patients whose clinical visits were assigned a combination of ICD-9 and ICD-10 codes that satisfied the Cleveland Clinic criteria. The complete EHR of these patients—comprising clinical notes, genetic reports, and billing codes—were extracted. At each site, the charts of a subset of these patients were reviewed by a team of clinical experts from that site in order to determine (A) if that patient indeed met Cleveland Clinic criteria, (B) if that patient had any genetic testing, (C) if the genetic testing included *PTEN* sequencing and/or deletion duplication analysis, and (D) if there was a likely pathogenic or pathogenic variant detected in *PTEN*.

### Determination of whether the patient satisfied the Cleveland Clinic criteria

The presence of macrocephaly was assumed to be true for all patients due to inconsistent documentation about head circumference or inability to ascertain this clinical feature. To determine if each patient satisfied Cleveland Clinic (CC) criteria (i.e., if the patient had *at least* one of the four additional clinical features mentioned in the criteria), the reviewing team iteratively evaluated each patient record to be reviewed using a protocol (detailed in Supplementary Methods under Protocol for determination of whether patient satisfied Cleveland Clinic criteria)

### Determination of whether the patient had genetic testing

To determine if the patient had genetic testing, the reviewing team followed a protocol (detailed in Supplementary Methods under "Protocol for determination of whether patient had genetic testing").

### Determination of whether genetic testing included *PTEN* analysis

If the patient had genetic testing, the reviewing team reviewed the list of genetic tests to identify inclusion of *PTEN* analysis. The following tests were among those automatically deemed to include *PTEN* analysis: *PTEN* single gene sequencing, *PTEN* deletion/duplication analysis, and whole exome sequencing. For gene panels, the team manually reviewed the report if available to determine if *PTEN* was included in the panel. If the report was not available, then the testing laboratory’s website was queried to see if *PTEN* was part of the panel. See Fig. S2.

### Determination of whether the patient had a pathogenic or likely pathogenic variant in *PTEN*

If the patient had genetic testing that included *PTEN* sequencing, the team reviewed the original report, or references to the test results, to see if there was a reported likely pathogenic or pathogenic variant in *PTEN*. If so, the patient was deemed to *have PHTS*. Otherwise, the patient was deemed to *not have PHTS*.

## Results

The yield—the number of patients who needed to be genetically tested for a pathogenic *PTEN* variant—of the Cleveland Clinic criteria ranged from 82 to 94% at the three centers (Table 2).

### Review of yield of informatics approach

#### Boston Children’s Hospital (BCH)

With this informatics approach, there were 1215 patients at Boston Children’s Hospital identified as having met Cleveland Clinic criteria. Human review of clinical documentation of 396 randomly selected patients was performed. Of these 396 patients, 371 patients did indeed satisfy Cleveland Clinic criteria (see Table 1). For the BCH site, the yield of this informatics approach in correctly identifying patients who met Cleveland Clinic criteria was 93.69%.

#### Children’s National Hospital (CNH)

With this informatics approach, there were 481 patients at Children’s National Hospital identified as having met Cleveland Clinic criteria. Human review of clinical documentation of all of these patients identified 438 patients as having truly met Cleveland Clinic criteria. For the CNH site, the yield of this informatics approach in correctly patients who met Cleveland Clinic criteria was 91.06%.

#### University of Washington (UW)

At the University of Washington, 94 patients were randomly selected for human review, out of the 104 patients who satisfied the Cleveland Clinic criteria using the informatics approach. After human review, 77 out of the 94 patients indeed satisfied the Cleveland Clinic criteria, resulting in a yield of 81.91%.

### Review of genetic testing

We also evaluated the number of patients who had molecular confirmation of the PHTS diagnosis. Among those patients who met Cleveland Clinic criteria identified by this informatics approach, and whose charts were reviewed, the percentage of patients with a molecular diagnosis of PHTS was 0.0% at UW, 2.7% at CNH, and 3.5% at BCH. Among those patients who met Cleveland Clinic criteria identified by this informatics approach, whose charts were reviewed, and who also had any genetic testing done which would have captured *PTEN* variants, this percentage is higher: 30.2% at CNH and 15.6% at BCH (Table 2).

## Discussion

Conditions associated with rare genetic diseases are largely underrepresented [26, 27] in commonly used clinical terminologies such as the ICD-10 and ICD-9. The problem persists in the latest version of the International Classification of Diseases (ICD-11) terminology [28], where conditions associated with genetic diseases are either categorized in counterintuitive ways, too broadly generalized, or not defined at all [29]. In this work, we have demonstrated the feasibility of using a computational phenotype across multiple institutions to identify patients who satisfy Cleveland Clinic criteria and who may therefore benefit from *PTEN* genetic analysis. The positive predictive value of this approach at each of the three sites exceeded 80%, suggesting that an informatics approach may be able to bypass the shortcoming of the ICD9/10 code system in explicitly including “PTEN hamartoma tumor syndrome.”

We also evaluated the percentage of patients who were correctly identified as having PHTS, out of the total number of patients identified as satisfying Cleveland Clinic criteria through this informatics approach. While this number was low across the three sites—between 0 and 3.5%—several factors account for this. First, the number of patients with PHTS identified may reflect the very low prevalence of PHTS, which according to one estimate is 1:200,000 [30]. Second, these percentages do not take into account those who did not undergo any genetic testing in the first place. Third, not every patient who underwent genetic testing had genetic testing that included *PTEN* sequencing.

These percentages become higher (15.6%, 30.2%) when the denominator is further limited by those who have undergone genetic testing which would have captured *PTEN* variants. It is worthwhile to compare this higher range of percentages (i.e., *PTEN* molecular diagnosis among those identified by informatics approach who had genetic testing that included detection of *PTEN* variants) to clinical scenarios reported in prior studies of diagnostic yield of *PTEN* testing in different cohorts. For example, in the original data serving as the basis for the Cleveland Clinic pediatric PTEN criteria, there were 92 pediatric patients who met relaxed International Cowden Consortium operational criteria for CS [23], of whom 28 had a *PTEN* mutation (30.4%). In a retrospective study of the percentage of patients with a confirmed *PTEN* mutation among different pediatric cohorts, 2/14 (14.2%) had PHTS among those with ASD and macrocephaly, 3/13 (23.1%) had PHTS among those with ASD and developmental delay/ID and macrocephaly, and 6/32 (18.8%) had PHTS among those with developmental delay/ID and macrocephaly [31]. Hence, the informatics approach used in our study not only shows promise in identifying those who may meet Cleveland Clinic PTEN criteria but also underscores that there were many patients who may have benefited from genetic testing but who did not actually undergo genetic testing. This is evident by the large percentage of patients in our study identified by informatics approach as having met Cleveland Clinical PTEN criteria, who either did not have genetic testing or had genetic testing which did not include analysis of *PTEN* variants.

The approach taken here across three academic research centers can be used at several other institutions around the country in the future to identify patients that would benefit from PTEN sequencing. Furthermore, similar computational phenotypes can be developed and tested for other rare genetic disorders. For example, if a clinician is evaluating a patient for whom only electronic health records are available, the use of a computational phenotype could help delineate a phenotype caused by a particular gene defect.

### Limitations

Limitations in this informatics approach for detecting patients who met Cleveland Clinic criteria for PTEN testing are evident in the instances of false positives, that is, those who met Cleveland Clinic criteria by the informatics approach but who on review of the medical records did not actually meet Cleveland Clinic criteria. A large contributing factor is that the billing codes may not accurately or completely encompass the clinical phenotype. In addition, there may be inaccuracies in the billing codes. For instance, in some cases, providers coded patients as having developmental delay, when the clinical documentation specifically mentioned “normal development.” There can be a mismatch in actual clinical information vs. intention behind billed ICD codes. For example, there was an instance in which a patient postoperatively lost speech but regained this ability later on. The provider coded this as expressive language disorder, perhaps because another more suitable billing code was not identifiable.

Coding systems such as ICD-10 and ICD-9 were developed primarily for administrative purposes [32]. Given the lack of precise clinical codes for genetic diseases and their symptoms, errors in coding can be difficult to avoid [33]. Studies have revealed widespread inconsistencies in the precision of billing codes in capturing clinical symptoms [34, 35]. In other words, though it is feasible to use billing codes to ascertain Cleveland Clinic criteria, there is a need for improved precision of clinical codes in capturing clinical phenotype diversity to address this limitation. Deep phenotyping [36, 37], using finer-grained representations of disease phenotypes as defined in terminologies such as the Human Phenotype Ontology (HPO) [38] and SNOMED CT [39], is essential for precise characterization and phenome-based diagnosis of rare diseases such as PHTS.

There were several additional limitations. First, we did not analyze whether patients identified as having met Cleveland Clinic criteria, and whose charts were reviewed and confirmed to meet Cleveland Clinic criteria, reported another clinical reason to suspect a diagnosis other than PHTS. Second, we did not ascertain whether macrocephaly was truly present, due to inconsistent availability and documentation of head circumference. This may help account for the low fraction of individuals who fulfill Cleveland Clinic criteria who have pathogenic *PTEN* variants. For example, at the BCH site, we identified an example of one patient with PHTS with macrocephaly and related dermatological findings who would have fulfilled Cleveland Clinic criteria, but macrocephaly was not billed as a diagnosis. Third, we did not limit EMR data to that *prior* to the diagnosis (given that a patient diagnosis would influence what clinical features are referenced in the notes), since it was not straightforward to ascertain age of diagnosis (though report date is one possibility, patient knowledge and provider knowledge of this diagnosis may lag). Finally, we did not have the data to evaluate race/ethnicity/social vulnerability index. On review of data from the BCH site, nearly 60% of the patients identified as having met Cleveland Clinic criteria using the informatics approach were white, suggesting that minorities were underrepresented, which limits generalizability. This point underscores continued need for attention to inclusion and diversity in ongoing research efforts, especially to the question of why minorities are underrepresented in research databases and clinical encounters.

## Conclusions

Computational phenotypes have the potential to greatly reduce the difficulties in diagnosing rare genetic disorders by identifying patient cohorts for genetic testing and also to enhance the clinical characterization of these diseases. In this paper, we have discussed the evaluation and effectiveness of a computational phenotype in identifying patients who need to be genetically screened for pathogenic *PTEN* variants from the EHR of patients. The observed yield of this computational phenotype results from the following: (A) the lack of emphasis on fine-grained representation of clinical symptoms in billing codes used at healthcare centers, (B) the slow pace of adoption of diagnostic methods based upon genetic testing into clinical practice, and (C) the limited understanding of the phenotypic diversity of genetic diseases.

However, the availability of genomic and phenotypic data from significantly larger patient populations and improvements in the representational capabilities of clinical terminologies in the long-term will greatly facilitate the drive towards precise clinical characterization of PHTS and its symptoms.

## Supplementary Information

**Additional file 1: Figure S1.** Workflow for the identification and validation of a computational phenotype for identifying patients who meet Cleveland Clinic criteria.

**Additional file 2: Figure S2.** Workflow for the review of genetic testing to determine if patients had a pathogenic variant in the *PTEN* gene that would confirm a molecular diagnosis of PHTS.

**Additional file 3: Supplementary Methods.** Protocol for determination of whether patient satisfied Cleveland Clinic criteria.

**Additional file 4: Table S1.** Billing Codes from ICD-9 and ICD-10 for Cleveland Clinical criteria for PHTS.

## Data Availability

The datasets used in this study are stored in the clinical data warehouses at the three centers: Boston Children’s Hospital, Children’s National Hospital, and the University of Washington. Because of patient privacy concerns, this data is not available to the general public. Requests for this data will need to be formally submitted to the Information Technology teams at these centers, and IRB clearances from the appropriate organizations will be needed as well. The code developed for this paper is available on GitHub. The ICD-9 and ICD-10 codes corresponding to the Cleveland Clinic criteria, which were used for searching the clinical data warehouses at Boston Children’s Hospital, Children’s National Hospital, and the University of Washington are listed in Table S1.

## Abbreviations

*PTEN*: Phosphatase and tensin homolog
PHTS: *PTEN* hamartoma syndrome
IDDRC: Intellectual and Developmental Disabilities Research Centers
BCH: Boston Children’s Hospital
CNH: Children’s National Hospital
EHR: Electronic health records
OMIM: Online Mendelian Inheritance in Man
HGNC: HUGO Gene Nomenclature Committee
NCBI: National Center for Biotechnology Information
ICD-9: International Classification of Diseases version 9
ICD-10: International Classification of Diseases version 10
UW: University of Washington
CC: Cleveland Clinic
IRB: Institutional Review Board
