# Supplementary figures and images for: Validation of a computational phenotype for finding patients eligible for genetic testing for pathogenic PTEN variants across three centers
Source: J Neurodev Disord. 2022 Mar 23;14:24. doi: 10.1186/s11689-022-09434-0 (PMC8943944; doi:10.1186/s11689-022-09434-0)

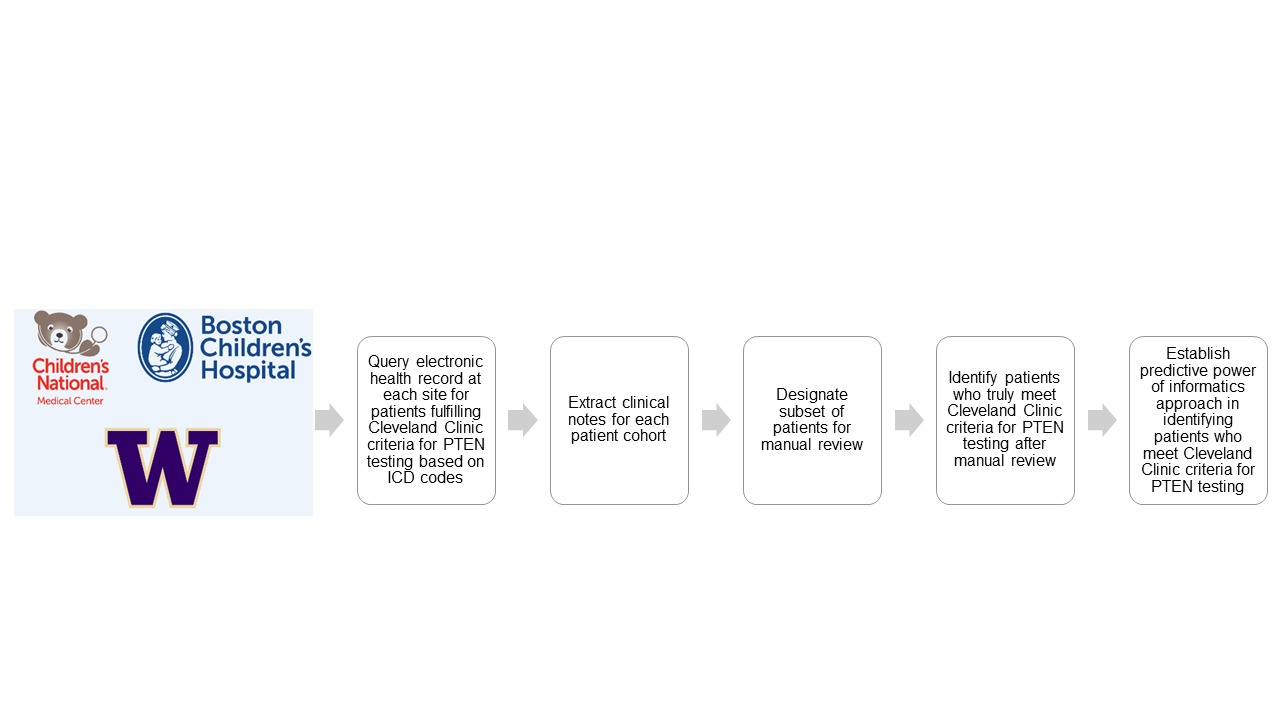

Supplement: Supplementary file 1 — Additional file 1: Figure S1. Workflow for the identification and validation of a computational phenotype for identifying patients who meet Cleveland Clinic criteria. [file 11689_2022_9434_MOESM1_ESM.tif]

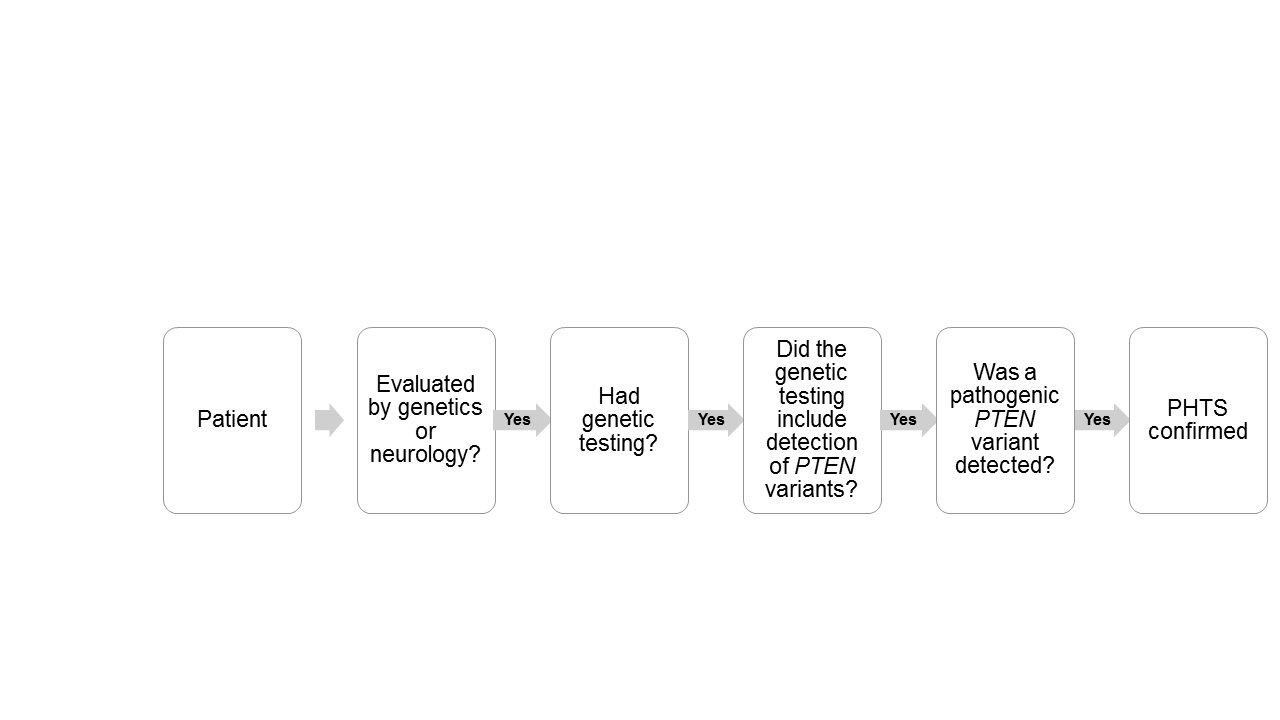

Supplement: Supplementary file 2 — Additional file 2: Figure S2. Workflow for the review of genetic testing to determine if patients had a pathogenic variant in the PTEN gene that would confirm a molecular diagnosis of PHTS. [file 11689_2022_9434_MOESM2_ESM.tif]
