# Supplementary material for: Validation of a computational phenotype for finding patients eligible for genetic testing for pathogenic PTEN variants across three centers
Source: J Neurodev Disord. 2022 Mar 23;14:24. doi: 10.1186/s11689-022-09434-0 (PMC8943944; doi:10.1186/s11689-022-09434-0)
Supplement: Supplementary file 3 — Additional file 3: Supplementary Methods. Protocol for determination of whether patient satisfied Cleveland Clinic criteria. [file 11689_2022_9434_MOESM3_ESM.docx]

**Supplementary Methods.**

**Protocol for determination of whether patient satisfied Cleveland Clinic criteria.**

- A. Is the latest outpatient neurology note available for review?
  - If no, go to step B
  - If yes, using this note, perform CC CORE STEPS #1-5.
    - If Cleveland Clinic criteria is not yet satisfied, go to step B.
- B. Is the latest outpatient genetics note available for review?
  - If no, go to step C.
  - If yes, using this note, perform CC CORE STEPS #1-5.
    - If Cleveland Clinic criteria is not yet satisfied, go to step C.
- C. Is the latest outpatient developmental medicine note available for review?
  - If no, go to step D.
  - If yes, using this note, perform CC CORE STEPS #1-5.
    - If Cleveland Clinic criteria is not yet satisfied, go to step D.
- D. Is the latest inpatient neurology note available for review?
  - If no, go to step E.
  - If yes, using this note, perform CC CORE STEPS #1-5.
    - If Cleveland Clinic criteria is not yet satisfied, go to step E.
- E. Is the latest inpatient genetics note available for review?
  - If no, go to step F.
  - If yes, using this note, perform CC CORE STEPS #1-5.
    - If Cleveland Clinic criteria is not yet satisfied, go to step F.
- F. Consider expanding note review to include other disciplines and perform CC CORE STEPS #1-5 for each type of note. If Cleveland Clinic is still not satisfied, then Cleveland Clinic is deemed to be *not satisfied* and STOP.
- CC CORE STEPS
  - #1. Determine if text from the note references an autism diagnosis (autism spectrum disorder, autism, Asperger syndrome, PDD-NOS, pervasive developmental disorder). If so, Cleveland Clinic criteria is *satisfied* and STOP. Otherwise go to step #2.
  - #2. Determine if text from the note references developmental delay (developmental delay, global developmental delay, global delay, intellectual disability, cognitive impairment, language delay, language disorder, speech disorder, communication disorder, expressive language disorder, mixed receptive expressive language disorder, social pragmatic language disorder, motor disorder, cerebral palsy, fine motor delay, gross motor delay). In cases where developmental delay is not explicitly stated, but where the patient receives speech/language therapy, physical therapy, or other developmental services, infer that developmental delay is present. If text from the note references developmental delay, or if developmental delay is inferred, Cleveland Clinic criteria is *satisfied* and STOP. Otherwise, go to step #3.
  - #3. Determine if text from the note references dermatological features (oral papillomas, lipomas, trichilemmomas, penile freckling). If so, Cleveland Clinic criteria is *satisfied* and STOP. Otherwise go to step #4.
  - #4. Determine if text from the note references vascular features (arteriovenous malformations, hemangiomas). If so, Cleveland Clinic criteria is *satisfied* and STOP. Otherwise go to step #5.
  - #5. Determine if text from the note references gastrointestinal features (gastrointestinal polyps). If so, Cleveland Clinic criteria is *satisfied* and STOP.

**Protocol for determination of whether patient had genetic testing.**

- A. Is the latest outpatient genetics note available for review?
  - If no, go to step B
  - If yes, using this note, perform GENETICS CORE STEP #1.
    - If determination of whether the patient had genetic testing is not yet made, go to step B.
- B. Is the latest outpatient neurology note available for review?
  - If no, go to step C.
  - If yes, using this note, perform GENETICS CORE STEP #1.
    - If determination of whether the patient had genetic testing is not yet made, go to step C.
- C. Is the latest outpatient developmental medicine note available for review?
  - If no, go to step D.
  - If yes, using this note, perform GENETICS CORE STEP #1.
    - If determination of whether the patient had genetic testing is not yet made, go to step D.
- D. Review the Labs section of the EMR to see if prior genetic testing was sent/resulted. Perform GENETICS CORE STEP #1.
  - If determination of whether the patient had genetic testing is not yet made, go to step E.
- E. Review the Outside Documentation section of EMR which contains scanned copies of outside laboratory results, focusing on documents that may have genetic test results. Perform GENETICS CORE STEP #1. If determination of whether the patient had genetic testing is still not yet made, then the patient is deemed to *not have had genetic testing.*
- GENETICS CORE STEP
  - #1. Determine if content summarizes or references prior genetic testing, including but not limited to, the following: chromosomal microarray, fragile X testing, single gene sequencing, gene panel, whole exome sequencing, and mitochondrial DNA sequencing. If so, the patient is deemed to *have had genetic testing*, and STOP. For each genetic test the patient has had, identify the name of the test, the test results, the testing laboratory, and a copy of the report if available.
