# Supplementary material for: Validation of a computational phenotype for finding patients eligible for genetic testing for pathogenic PTEN variants across three centers
Source: J Neurodev Disord. 2022 Mar 23;14:24. doi: 10.1186/s11689-022-09434-0 (PMC8943944; doi:10.1186/s11689-022-09434-0)
Supplement: Supplementary file 4 — Additional file 4: Table S1. Billing Codes from ICD-9 and ICD-10 for Cleveland Clinical criteria for PHTS. [file 11689_2022_9434_MOESM4_ESM.docx]

**Table S1.** Billing Codes from ICD-9 and ICD-10 for Cleveland Clinical criteria for PHTS

| **CONDITIONS IN Cleveland Clinic REPORT** | **ICD-10 Code** | **ICD-10 Label** | **ICD-9 Code** | **ICD-9 Label** |
| --- | --- | --- | --- | --- |
| Macrocephaly (>= 2SD) | Q75.3 | Macrocephaly | NA | NA |
| AND one of ( |  |  |  |  |
| Autism | F84.0 | Autistic Disorder | 299.00 | Autistic Disorder Current or Active State |
|  | F84.5 | Asperger's Syndrome | 299.01 | Autistic Disorder Residual State |
|  | F84.9 | Pervasive Development Disorder, Unspecified |  |  |
|  | F84.8 | Other Pervasive Development Disorders |  |  |
| *OR* |  |  |  |  |
| Developmental Delay | F80 | Specific Developmental Disorders of Speech and Language | 315.31 | Expressive Language Disorder |
|  | F80-F89 | Pervasive and Specific Developmental Disorders | 315.32 | Mixed Receptive Expressive Language Disorder |
|  | F80.1 | Expressive Language Disorder | 315.39 | Other Developmental Speech or Language Disorder |
|  | F80.2 | Mixed Receptive Expressive Language Disorder | 315.9 | Unspecified Delay in Development |
|  | F80.8 | Other Developmental Disorders of Speech and Language | 317 | Mild Intellectual Disabilities |
|  | F80.82 | Social Pragmatic Communication Disorder | 318.0 | Moderate Intellectual Disabilities |
|  | F80.89 | Other Developmental Disorders of Speech and Language | 318.1 | Severe Intellectual Disabilities |
|  | F80.9 | Developmental Disorder of Speech and Language Unspecified | 318.2 | Profound Intellectual Disabilities |
|  | F82 | Specific Developmental Disorder of Motor Function | 319 | Unspecified Intellectual Disabilities |
|  | F88 | Other disorders of psychological development |  |  |
|  | F89 | Unspecified Disorder of Psychological Development |  |  |
|  | R41.83 | Borderline Intellectual Functioning |  |  |
|  | R47 | Speech Disturbances Not Elsewhere Classified |  |  |
|  | R62.0 | Delayed Milestone in Childhood |  |  |
|  | R62.50 | Unspecified lack of expected normal physiological development in childhood |  |  |
|  | F70-F79 | Intellectual Disabilities |  |  |
| *OR* |  |  |  |  |
| Dermatological features including Lipomas, Trichillemomma, Oral Papilloma, and Penile Freckling | D10.3 | Benign neoplasm of other and unspecified parts of mouth | 210.4 | Benign neoplasm of other and unspecified parts of mouth |
|  | D10.30 | Benign neoplasm of unspecified part of mouth | 214.1 | Lipoma of Other Skin and Subcutaneous Tissue |
|  | D10.39 | Benign neoplasm of other parts of mouth | 214.2 | Lipoma of Intrathoracic Organs |
|  | D17.0 | Benign lipomatous neoplasm of skin and subcutaneous tissue of head, face, and neck | 214.3 | Lipoma of Intra-abdominal Organs |
|  | D17.1 | Benign lipomatous neoplasm of skin and subcutaneous tissue of trunk | 214.4 | Lipoma of Spermatic Cord |
|  | D17.20 | Benign lipomatous neoplasm of skin and subcutaneous tissue of unspecified limb | 214.8 | Lipoma of other specified sites |
|  | D17.21 | Benign lipomatous neoplasm of skin and subcutaneous tissue of right arm | 214.9 | Lipoma unspecified site |
|  | D17.22 | Benign lipomatous neoplasm of skin and subcutaneous tissue of left arm | 216.3 | Benign neoplasm of skin of other and unspecified parts of face |
|  | D17.23 | Benign lipomatous neoplasm of skin and subcutaneous tissue of right leg |  |  |
|  | D17.24 | Benign lipomatous neoplasm of skin and subcutaneous tissue of left leg |  |  |
|  | D17.30 | Benign lipomatous neoplasm of skin and subcutaneous tissue of unspecified sites |  |  |
|  | D17.39 | Benign lipomatous neoplasm of skin and subcutaneous tissue of other sites |  |  |
|  | D17.4 | Benign lipomatous neoplasm of skin and subcutaneous tissue of intrathoracic organs |  |  |
|  | D17.5 | Benign lipomatous neoplasm of skin and subcutaneous tissue of intra-abdominal organs |  |  |
|  | D17.6 | Benign lipomatous neoplasm of skin and subcutaneous tissue of spermatic cord |  |  |
|  | D17.71 | Benign lipomatous neoplasm of kidney |  |  |
|  | D17.72 | Benign lipomatous neoplasm of other genitourinary organs |  |  |
|  | D17.79 | Benign lipomatous neoplasm of other sites |  |  |
|  | D17.9 | Benign lipomatous neoplasm unspecified |  |  |
|  | D23.3 | Other benign neoplasm of skin of other and unspecified parts of face |  |  |
|  | D23.30 | Other benign neoplasm of skin of unspecified part of face |  |  |
|  | D23.39 | Other benign neoplasm of skin of other parts of face |  |  |
| *OR* |  |  |  |  |
| Vascular Features like Hemangiomas and Arteriovenous Malformations | D18.0 | Hemangioma | 228.00 | Hemangioma of unspecified site |
|  | D18.00 | Hemangioma unspecified site | 228.01 | Hemangioma of skin and subcutaneous tissue |
|  | D18.01 | Hemangioma of skin and subcutaneous tissue | 228.02 | Hemangioma of intracranial structures |
|  | D18.02 | Hemangioma of intracranial structures | 228.03 | Hemangioma of retina |
|  | D18.03 | Hemangioma of intra-abdominal structures | 228.04 | Hemangioma of intra-abdominal structures |
|  | D18.09 | Hemangioma of other sites | 228.09 | Hemangioma of other sites |
|  | Q25.7 | Other congenital malformations of pulmonary artery | 747.32 | Pulmonary arteriovenous malformation |
|  | Q25.72 | Congenital pulmonary arteriovenous malformation | 747.63 | Upper limb vessel anomaly |
|  | Q25.79 | Other congenital malformations of pulmonary artery | 747.64 | Lower limb vessel anomaly |
|  | Q27 | Other congenital malformations of peripheral vascular system | 747.81 | Anomalies of cerebrovascular system |
|  | Q27.2 | Other congenital malformations of renal artery |  |  |
|  | Q27.3 | Arteriovenous malformation (peripheral) |  |  |
|  | Q27.30 | Arteriovenous malformation, site unspecified |  |  |
|  | Q27.31 | Arteriovenous malformation of vessel of upper limb |  |  |
|  | Q27.32 | Arteriovenous malformation of vessel of lower limb |  |  |
|  | Q27.33 | Arteriovenous malformation of digestive system vessel |  |  |
|  | Q27.34 | Arteriovenous malformation of renal vessel |  |  |
|  | Q27.39 | Arteriovenous malformation, other site |  |  |
|  | Q27.9 | Congenital malformation of peripheral vascular system, unspecified |  |  |
|  | Q28 | Other congenital malformations of circulatory system |  |  |
|  | Q28.0 | Arteriovenous malformation of precerebral vessels |  |  |
|  | Q28.1 | Other malformations of precerebral vessels |  |  |
|  | Q28.2 | Arteriovenous malformation of cerebral vessels |  |  |
|  | Q28.3 | Other malformations of cerebral vessels |  |  |
|  | Q28.8 | Other specified congenital malformations of circulatory system |  |  |
|  | Q28.9 | Congenital malformation of circulatory system, unspecified |  |  |
| *OR* |  |  |  |  |
| Gastrointestinal Polyps | D12.6 | Benign neoplasm of colon unspecified | 211.3 | Benign neoplasm of colon |
|  | K31.7 | Polyp of stomach and duodenum | 569.0 | Anal and rectal polyp |
|  | K51.4 | Inflammatory polyps of colon |  |  |
|  | K51.40 | Inflammatory polyps of colon without complications |  |  |
|  | K51.41 | Inflammatory polyps of colon with complications |  |  |
|  | K51.411 | Inflammatory polyps of colon with rectal bleeding |  |  |
|  | K51.412 | Inflammatory polyps of colon with intestinal obstruction |  |  |
|  | K51.413 | Inflammatory polyps of colon with fistula |  |  |
|  | K51.414 | Inflammatory polyps of colon with abscess |  |  |
|  | K51.418 | Inflammatory polyps of colon with other complications |  |  |
|  | K51.419 | Inflammatory polyps of colon with unspecified complications |  |  |
|  | K62.0 | Anal polyp |  |  |
|  | K62.1 | Rectal polyp |  |  |
|  | K63.5 | Polyp of colon |  |  |
|  | V12.72 | Personal history of colonic polyps |  |  |
|  | Z86.010 | Personal history of colonic polyps |  |  |
| ) |  |  |  |  |
